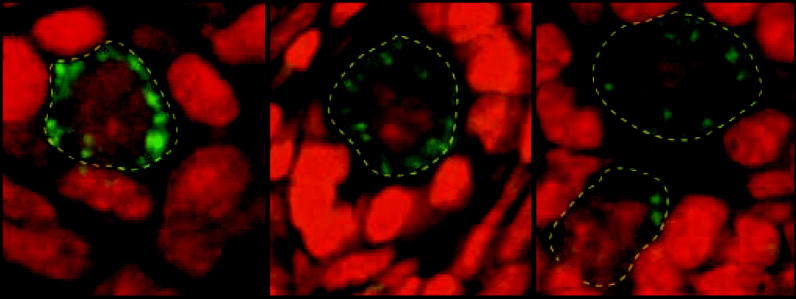# Headliners: Reproduction: Oocyte Generation in Adult Mice

**Published:** 2005-12

**Authors:** Jerry Phelps

Johnson J, Bagley J, Skaznik-Wikiel M, Lee H-J, Adams GB, Niikura Y, Tschudy KS, Tilly TC, Cortes ML, Forkert R, Spitzer T, Iacomini J, Scadden DT, Tilly JL. 2005. Oocyte generation in adult mammalian ovaries by putative germ cells in bone marrow and peripheral blood. Cell 122:303–315.

The theory that female mammals are born with a finite number of germ cells (oocytes) has been accepted as an unquestionable truth for over 50 years. Recent research has challenged this accepted dogma by showing that mice and flies can produce oocytes and follicles during puberty and adulthood. Now NIEHS grantee Jonathan L. Tilly and colleagues at the Harvard Medical School have shown that adult mice can produce large numbers of new oocytes in a short period of time, providing additional evidence to challenge the accepted belief of a fixed complement of oocytes at birth. The Harvard researchers also discovered a source of germline stem cells in the bone marrow.

Oocytes are found in the ovaries surrounded by somatic cells in structures known as follicles. Only a small fraction of follicles actually reach ovulation, producing an egg capable of being fertilized. Conventional wisdom hold that in humans, only about 30,000 of an original pool of about 1 million oocytes present at birth are still present at puberty, and this number is thought to gradually decline throughout adulthood until the complete loss of oocytes at around age 50 stimulates menopause. Acceptance of the concept that adult mammals can continue to produce oocytes has been slow, likely due to the lack of direct evidence of the existence of mammalian female germline stem cells.

The Harvard team conducted gene expression analysis and bone marrow transplantation studies on mice that had been sterilized through chemotherapy. Within 24 hours of treatment, follicles were regrowing in the animals’ ovaries. By 2 months after treatment, there was no difference between the treated animals and controls. In other studies, mice whose bone marrow was destroyed with chemotherapeutic agents were injected with peripheral blood from transgenic animals with germline cells expressing green fluorescent protein. Oocytes found in the test animals’ ovaries within 30 hours of treatment also expressed the fluorescent protein.

The researchers have not yet determined whether oocytes derived from germline stem cells can undergo fertilization and subsequently develop into viable offspring. However, the results do prove that bone marrow and peripheral blood are sources of germline stem cells and can sustain oocyte production into adulthood. If adult oocyte production is also possible in humans, it could have major implications for the treatment of infertility and other disorders such as osteoporosis, although much additional research is needed before this potential can be realized.

## Figures and Tables

**Figure:**